# Is background methotrexate advantageous in extending TNF inhibitor drug survival in elderly patients with rheumatoid arthritis? An analysis of the British Society for Rheumatology Biologics Register

**DOI:** 10.1093/rheumatology/kez671

**Published:** 2020-01-30

**Authors:** Katie Bechman, Anuoluwapo Oke, Mark Yates, Sam Norton, Elaine Dennison, Andrew P Cope, James B Galloway

**Affiliations:** 1 Centre for Rheumatic Diseases, Kings College London, LondonUK; 2 MRC Lifecourse Epidemiology Unit, University of Southampton, Southampton, UK; 3 Psychology Department, Institute of Psychiatry, Kings College London, London, UK

**Keywords:** rheumatoid arthritis, biologics, anti-TNF therapy, methotrexate, epidemiology

## Abstract

**Objective:**

To evaluate drug survival with monotherapy compared with combination therapy with MTX in RA older adults.

**Methods:**

Patients from the British Society for Rheumatology Biologics Register, a prospective observational cohort, who were biologic naïve and commencing their first TNF inhibitors (TNFi) were included. The cohort was stratified according to age: <75 and ≥75. Cox-proportional hazards models compared the risk of TNFi discontinuation from (i) any-cause, (ii) inefficacy and (iii) adverse events, between patients prescribed TNFi-monotherapy compared with TNFi MTX combination.

**Results:**

The analysis included 15 700 patients. Ninety-five percent were <75 years old. Comorbidity burden and disease activity were higher in the ≥75 cohort. Fifty-two percent of patients discontinued TNFi therapy during the follow-up period. Persistence with therapy was higher in the <75 cohort. Patients receiving TNFi monotherapy were more likely to discontinue compared with patients receiving concomitant MTX [hazard rate 1.12 (1.06–1.18) *P *<0.001]. This finding only held true in patients <75 [hazard rate (HR) 1.11 (1.05–1.17) *vs* ≥75 [HR 1.13 (0.90–1.41)]. Examining TNFi discontinuation by cause revealed patients ≥75 receiving TNFi monotherapy were less likely to discontinue TNFi due to inefficacy [HR 0.66 (0.43–0.99) *P*=0.04] and more likely to discontinue therapy from adverse events [HR 1.41(1.02–1.96) *P *=0.04]. These results were supported by the multivariate adjustment in complete case and imputed analyses.

**Conclusion:**

TNFi monotherapy is associated with increased treatment failure. In older adults, the disadvantage of TNFi monotherapy on drug survival is no longer seen. Patients ≥75 have fewer discontinuations due to inefficacy than adverse events compared with younger patients. This likely reflects greater disposition to toxicity but perhaps also a decline in immunogenicity associated with immunosenescence.

**Rheumatology key messages**

- TNFi monotherapy is associated with increased treatment failure in RA.
- In RA patients ≥75, the disadvantage of TNFi monotherapy on drug survival is no longer seen.
- Older RA patients have fewer discontinuations due to inefficacy compared with younger patients.

## Introduction

In the management of RA, MTX continues to serve as the ‘anchor drug’, demonstrating efficacy as a first-line therapy and is established as the standard of care worldwide [1]. Biologics are routinely used in patients who have failed treatment with MTX and/or other conventional synthetic disease-modifying antirheumatic drugs (csDMARDs). Current national UK guidelines advocate administering biologics in combination with MTX therapy for those patients with an inadequate response to csDMARDs alone.

Randomized controlled trial data consistently demonstrate superior efficacy in controlling disease activity with TNF blockade in combination with MTX over TNF inhibitors (TNFi) monotherapy [2–7]. Longer-term observational data from national registries allow the examination of treatment continuation rates (drug survival). Drug survival is influenced by various factors including lack or loss of clinical efficacy, adverse events and poor adherence. Despite a good initial response to a TNF inhibitor, efficacy can wane over time. Secondary failure may result from the formation of antidrug antibodies generated as a consequence of an immune response to the protein base agent, potentially neutralizing its therapeutic effect. Concomitant immunosuppression with MTX has a synergistic advantage. MTX increases TNFi concentrations via the suppression of antidrug antibodies, prolonging TNFi drug survival [8].

Registry data suggest superior drug survival with TNFi MTX combination compared with TNFi monotherapy [9–11]. A systematic review of published data from European and non-European registries reported that TNFi/csDMARD combinations reduced the risk of discontinuations from lack of efficacy [12]. Individual registries also describe superior survival rates with TNFi/csDMARD combinations, driven by fewer terminations from adverse events [13].

Adults aged over 65 years old are under-represented in RA clinical trials and data mainly originates from *post hoc* analyses. Whilst the efficacy and safety of TNF blockade in patients over 65 years has been examined in observational studies, the results are conflicting [14–19]. Some report reduced efficacy of TNFi in the elderly [14, 18] whilst other studies have not demonstrated an association with age and treatment response [15, 17] or rates of TNFi discontinuation [16]. The reasons for TNFi discontinuation may differ depending on age, with older patients discontinuing more frequently as a result of adverse events and younger patients as a result of inefficacy [17, 20].

Older age may associate with a reduction in the immunogenicity of biologic therapies. The aging immune system undergoes a gradual process of decline, termed immunosenescence. This affects both the innate and adaptive arms of the immune response. Key feature includes the suppression of phagocytosis by neutrophils and macrophages, altered cytokine production and a decrease in number and function of T and B lymphocytes and NK cells [21–26]. T-cell diversity is maintained in patients up to 65 years of age, despite thymic output ceasing by ∼50. After this, there is a rapid loss of clonal heterogeneity in individuals aged 75–80 years, with the T-cell repertoire diversity a mere 1% that of a younger cohort [27]. With increasing age there are important changes in antibody diversity with a decline in the ability to produce specific antibodies [24]. It is plausible that the production of antidrug antibodies that neutralize the effect of TNF inhibitors is less robust in elderly adults, reducing the risk of secondary failure and eliminating the need for concomitant immunosuppression.

The primary objective of this study was to investigate drug survival rates with TNFi monotherapy compared with combination therapy with MTX in older adults. We hypothesise that TNFi drug survival is different in these patients and the use of combination therapy might not prove as advantageous in older adults as it is in the younger cohort.

## Methods

### Patient population

Patients in this analysis were participants in the British Society of Rheumatology Biologics Register for RA (BSRBR-RA), a national prospective observational cohort study established in 2001 to monitor long-term safety of biological therapy. The BSRBR-RA methodology has been described previously [28]. Ethical approval was granted in 2000 [North West MREC (Multicentre Research Ethics Committee), reference 00/8/053]. Data uploaded to the BSRBR-RA by June 2016 were included in this analysis. All patients with RA who were biologic naïve and commencing their first TNF inhibitors (infliximab, etanercept, adalimumab and certolizumab) were eligible for inclusion in the analysis. The initial BSRBR-RA biologic cohorts in 2001 were for etanercept and infliximab users. Adalimumab and certolizumab-pegol cohorts were recruited later. A golimumab cohort has not been recruited. We chose a cut-off in age at 75 years *a priori* for the primary analysis for pragmatic reasons. Previously analyses have used an age of 65, although this is probably too young to anticipate a difference attributable to immunosenescence. Due to diminishing sample sizes it would have been inappropriate to select a sample any higher than 75 years. Our exploratory analyses have considered other age cut-off points (Supplementary Table S1, available at *Rheumatology* online).

### Baseline data

At registration, baseline data included demographics, comorbidity, smoking status, RA disease duration, RA disease activity (28-joint count Disease Activity Score), HAQ and csDMARD and corticosteroid exposure. Comorbidities were obtained from the patient’s medical records, using a pre-specified list of coexisting conditions. Comorbidity burden was scored using the Rheumatic Disease Comorbidity Index (RDCI), composed of 11 weighted past or present comorbid conditions. The RDCI performs well in predicting RA specific outcomes including disability, medical costs, hospitalisation and death [29–31].

### Follow-up

Follow-up data were collected every 6 months for the first 3 years by questionnaires sent to patients and their supervising rheumatology teams, and annually thereafter by questionnaires sent to the supervising rheumatology team only. Data on adverse events were captured from clinician questionnaires, from 6-monthly patient diaries detailing new hospital admissions, and by linkage to NHS Digital, which provides mortality data. NHS Digital has near complete capture of mortality data in the UK as all deaths (irrespective of where the death occurs) are centrally registered.

### Outcome

The primary outcome was persistence with first TNFi therapy, which was defined as the duration of time the patients continued to receive TNF blockade. Individuals were considered ‘at risk’ from treatment start for 5 years, or until treatment stop date, date of the last follow-up or date of death, whichever came first. Temporary stops of <90 days, after which the patient restarted the same anti-TNF therapy were counted as continuous use of the drug. Secondary outcomes included reason for TNF discontinuation separated according to inefficacy and adverse events.

### Statistical analysis

The cohort was divided according to age at registration: <75 and ≥75 years. Baseline characteristics were tabulated and tested for statistically significant imbalance using χ^2^, Mann–Whitney or t-tests, as appropriate. Kaplan–Meier survival curves were used to describe the persistence with anti-TNF therapy. The incidence rate (IR) of treatment discontinuation was calculated per 100 patient-years with 95% CI. Cox proportional hazards models were used to compare the risk of TNFi discontinuation between patients prescribed TNFi monotherapy compared with those receiving TNFi MTX combination (the reference group). Three models were developed, evaluating treatment discontinuation: (i) any cause, (ii) inefficacy and (iii) adverse events. For the separate inefficacy and adverse event analyses, a competing risk survival model was used following the Fine & Gray method allowing for accurate estimates of cumulative incidence [32]. Multivariable adjustment was made for the following baseline covariates: age, sex, disease duration, DAS28, HAQ, RDCI, smoking status and steroid exposure.

Baseline missing data were addressed using multiple imputation, with multivariate sequential imputation using chained equations for 20 imputations (Supplementary Table S2, available at *Rheumatology* online). The HAQ-DI was analysed as a continuous variable. We did not have access to item-level data for the HAQ-DI to Rasch transform it. We used predictive mean matching approach in the imputation model to account for this.

To address confounding by indication, a sensitivity analysis was performed using a propensity score (PS) model employing inverse probability of treatment weights for patients receiving TNFi monotherapy compared with those receiving TNFi MTX combination. The PS model included the following baseline covariates: age, sex, disease duration, DAS28, HAQ, RDCI, smoking status and steroid exposure (Supplementary Table S3, available at *Rheumatology* online).

Further analyses compared TNFi discontinuation in patients prescribed TNFi with other csDMARDs combinations. All analyses were undertaken using Stata 15 (StataCorp., College Station, TX, USA).

## Results

### Patient characteristics

Of 23 411 subjects registered in the BSRBR-RA, 15 700 were biologic naïve and commencing their first TNF inhibitor. Ninety-five percent of the cohort were younger than 75 years old. Overall mean age was 55 (s.d. 12.9), with a median disease duration of 10 years [interquartile range (IQR) 5–18]. Baseline mean DAS-28 was 6.42 (s.d. 1.06), reflective of a UK biologic initiation cohort. Baseline characteristics are in Table 1.

**Table 1:** Baseline characteristics by age group

	<75 years old	≥75 years old	Stat. imbalance
Total cohort, *n* (%)	14 932 (95.1)	768 (4.9)	
Age, yrs,	55 (46–63)	77 (76–80)	
Female sex, *n* (%)	10 788 (72.3)	627 (81.6)	<0.001^b^
Smoking status, *n* (%)			
Current	2648 (22.3)	43 (7.0)	<0.001^b^
Ever	7597 (61.6)	393 (60.3)	0.53^b^
Comorbidity (RDCI score ≥1), *n* (%)	8303 (55.6)	551 (71.7)	<0.001^b^
Cardiac (MI, stroke, angina)	968 (6.5)	133 (17.3)	<0.001^b^
Respiratory (asthma, COPD)	2080 (13.9)	129 (16.9)	<0.03^b^
Seropositive (RF), *n* (%)	8437 (58.7)	485 (64.3)	<0.002^b^
Disease duration, yrs	10 (5–18)	14 (7–23)	<0.0001^a^
Number of previous csDMARDs	3 (2–5)	3 (2–5)	0.33^a^
TNFi, *n* (%)			0.82^b^
Infliximab	3955 (26.5)	209 (27.2)	
Etanercept	5374 (36.0)	265 (34.5)	
Adalimumab	4744 (31.8)	246 (32.0)	
Certolizumab	859 (5.8)	48 (6.3)	
TNFi monotherapy, *n* (%)	3642 (24.4)	268 (34.9)	<0.001^b^
TNFi/csDMARDs combination			
Methotrexate	5776 (38.7)	252 (33.8)	
Sulfasalazine	430 (2.9)	21 (2.7)	
Leflunomide	667 (4.5)	43 (5.6)	
Two csDMARDs	2930 (19.6)	111 (14.5)	
Three csDMARDs	781 (5.2)	30 (3.9)	
Other combination	706 (4.7)	43 (5.6)	
Prednisolone, *n* (%)	5867 (39.3)	401 (52.2)	<0.001^b^
DAS28-ESR, mean (s.d.)	6.42 (1.1)	6.52 (1.0)	0.01^a^
SJC28, mean (s.d.)	10.7 (6.2)	10.6 (6.0)	0.84^a^
TJC28, mean (s.d.)	15.2 (7.5)	15.1 (7.9)	0.67^a^
Global VAS	75 (62-87)	75 (60–87)	0.20^a^
ESR	38 (21–61)	43 (26–68)	<0.0001^a^
CRP mg/l	26 (11–56)	29 (13–60)	0.12^a^
HAQ, median (IQR)	2.125 (1.625-2.375)	2.25 (2–2.625)	<0.0001

All values are gives as median (IQR), unless otherwise specified by n (%) or mean (s.d.).

a Statistical imbalance tested by kwallis.

b Statistical imbalance tested by χ^2^.

TNFi: TNF inhibitor; RDCI: Rheumatic Disease Comorbidity Index; DAS28: Disease Activity Score 28 Joints; SJC28: 28 swollen joint count; TJC28: 28 tender joint count; Global VAS: visual analogue scale for patient’s global assessment.

### Patients 75 years and older

As expected, the ≥75 cohort demonstrated greater comorbidity burden compared with the younger cohort (RDCI score ≥1 in 72% *vs* 56%, *P *< 0.001), with a higher prevalence of both cardiac and respiratory disease. RA disease activity measured by DAS28-ESR was higher in the ≥75 cohort (mean DAS28 6.52 *vs* 6.42, *P *= 0.009). This was driven by a higher ESR [median 43 (IQR 26–68) *vs* 38 (21–61), *P *< 0.0001] with no significant difference in the number of tender and swollen joints or global visual analogue scale (VAS) between the two age groups. A greater proportion of the ≥75 cohort were prescribed prednisolone (52% *vs* 39%, *P *< 0.001); however, there was no difference in the number of previous csDMARDs or choice of TNFi agents. Older patients were more likely to be prescribed TNFi monotherapy over combination with csDMARDs (35% *vs* 24%, *P *< 0.0001).

Seventy-five percent of patients were prescribed TNFi in combination with csDMARDs, rather than as monotherapy. There were several key differences comparing patients on TNFi monotherapy to combination therapy; patients on TNFi monotherapy demonstrated greater comorbidity burden, elevated markers of RA disease activity and disability, and a higher number of previous failed csDMARDs and concurrent prednisolone exposure (Supplementary Table S4, available at *Rheumatology* online).

### Persistence of TNF blockade

Fifty-two percent of the cohort (*n* = 8206) discontinued their first TNFi therapy during the follow-up period. With 44 642 person-years follow-up, the overall incidence of discontinuation was 18.4 (95% CI: 18.0, 18.8) per 100 patient years. Major reasons for discontinuation were adverse event (40%) and inefficacy (41%).

**Fig. 1:**
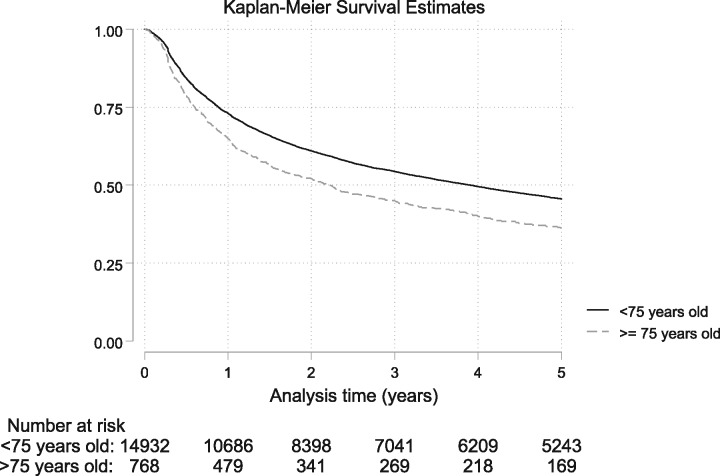
Kaplan–Meier estimates of crude persistence with TNFi therapy by age group

Persistence with TNFi therapy was higher in the younger cohort (Fig. 1). The crude IRs per 100 patient years for TNFi discontinuation were higher in the ≥75 compared with <75 age group; all cause: IR 25.5 (95% CI: 23.2, 27.9) *vs* IR 18.1 (95% CI: 17.7, 18.5), inefficacy: IR 8.4 (95% CI: 7.2, 9.9) *vs* IR 7.4 (95% CI: 7.2, 7.7) and adverse events: IR 11.8 (95% CI: 10.3, 13.6) *vs* IR 7.1 (95% CI: 6.9, 7.4) (Table 2).

**Table 2:** Incidence rate and Cox proportional hazard estimates (95% CI) for anti-TNF therapy discontinuation

	Age		
	<75 yrs (*n* = 14 932)	≥75 yrs (*n* = 768)	Total
Number of subjects	14 932	768	15 700
Patients with TNFi failure, *n* (% of cohort)	7756 (51.9)	450 (58.6)	8206 (52.3)
Reason for TNFi failure, *n* (%)			
Inefficacy	3193 (41.2)	149 (33.1)	3342 (40.7)
Adverse effect	3044 (39.3)	209 (46.4)	3253 (39.6)
Remission	51 (0.07)	5 (1.1)	56 (0.7)
Other	1171 (15.1)	75 (16.7)	1246 (15.2)
Missing	297 (3.8)	12 (2.7)	309 (3.8)

TNFi failure – all cause			
Follow up (person-years)	42 876	1766	44 642
No. of TNFi patients with TNFi failures	7756	450	8206
Incidence rate / 100 patient years (95% CI)	18.1 (17.7, 18.5)	25.5 (23.2, 27.9)	18.4 (18.0, 18.8)
Hazard ratio (95% CI) (ref methotrexate)			
Unadjusted; monotherapy	1.11 (1.05, 1.17)*	1.13 (0.90, 1.41)	1.12 (1.06, 1.18)*
Adjusted (imputed); monotherapy	1.07 (1.01, 1.13)∗∗	1.15 (0.91, 1.45)	1.08 (1.02, 1.14)∗∗
Propensity (imputed); monotherapy	1.06 (1.00, 1.12)	1.12 (0.90, 1.40)	1.06 (1.01, 1.13)∗∗

TNFi failure – inefficacy			
Follow up (Person-years)	42 876	1766	44 642
No. of TNFi patients with TNFi inefficacy	3193	149	3342
Incidence rate / 100 patient years (95% CI)	7.4 (7.2, 7.7)	8.4 (7.2, 9.9)	7.5 (7.2, 7.7)
Hazard ratio (95% CI) (ref methotrexate)			
Unadjusted; monotherapy	1.06 (0.97, 1.16)	0.66 (0.43, 0.99)∗∗	1.03 (0.95, 1.13)
Adjusted (imputed); monotherapy	1.06 (0.97, 1.16)	0.63 (0.41, 0.97)∗∗	1.03 (0.94, 1.13)
Propensity (imputed); monotherapy	1.06 (0.97, 1.16)	0.69 (0.45, 1.04)	1.04 (0.95, 1.13)

TNFi failure – adverse events			
Follow up (person-years)	42 876	1766	44 642
No. of TNFi patients with TNFi inefficacy	3044	209	3253
Incidence rate / 100 patient years (95% CI)	7.1 (6.9, 7.4)	11.8 (10.3, 13.6)	7.3 (7.0, 7.5)
Hazard ratio (95% CI) (ref MTX)			
Unadjusted; monotherapy	1.21 (1.11, 1.32)*	1.41 (1.02, 1.96)∗∗	1.23 (1.13, 1.34)*
Adjusted (imputed); monotherapy	1.13 (1.03, 1.23)*	1.46 (1.05, 2.03)∗∗	1.14 (1.05, 1.25)*
Propensity (imputed); monotherapy	1.11 (1.02, 1.22)*	1.35 (0.97, 1.88)	1.13 (1.04, 1.23)*

Adjusted for age, gender, disease duration, Rheumatic Disease Comorbidity Index, smoking, DAS28, HAQ-DI and steroid use.

Reference group = TNFi MTX combination. Supplementary Table S5, available at *Rheumatology* online reports on the hazard estimates for TNFi discontinuation by choice of combination therapy including TNFi-sulfasalazine, TNFi-leflunomide or TNF-multiple csDMARDs.

* *P*-value <0.01,

** *P*-value <0.05.

TNFi: TNF inhibitor.

**Fig. 2:**
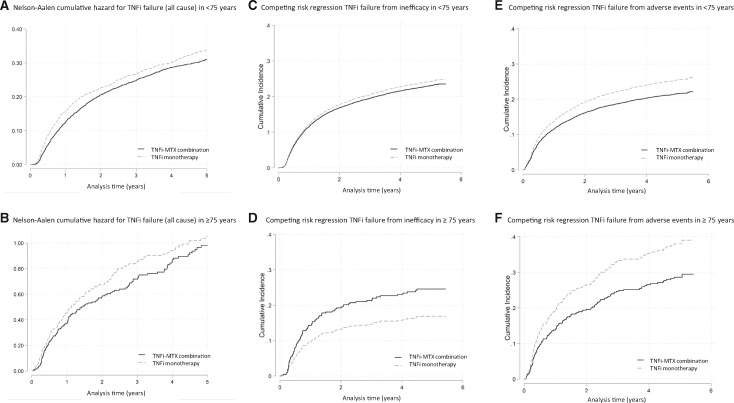
Cumulative hazard estimates of TNFi failure in patients on TNFi monotherapy and TNFi MTX combination therapy, by cause and by age (**A**) Nelson-Aalen cumulative hazard for TNFi failure from all cause in patients <75 years old on TNFi monotherapy and TNFi MTX combination therapy. (**B**) Nelson-Aalen cumulative hazard for TNFi failure from all cause in patients ≥75 years old on TNFi monotherapy and TNFi MTX combination therapy. (**C**) Competing risk regression with cumulative incidence of TNFi failure from inefficacy in patients <75 years old on TNFi monotherapy and TNFi MTX combination therapy. (**D**) Competing risk regression with cumulative incidence of TNFi failure from inefficacy in patients ≥75 years old on TNFi monotherapy and TNFi MTX combination therapy. (**E**) Competing risk regression with cumulative incidence of TNFi failure from adverse events in patients <75 years old on TNFi monotherapy and TNFi MTX combination therapy. (**F**) Competing risk regression with cumulative incidence of TNFi failure from adverse events in patients ≥75 years old on TNFi monotherapy and TNFi MTX combination therapy.

Overall, patients receiving TNFi monotherapy were more likely to discontinue TNF blockade compared with patients receiving TNFi MTX combination therapy [hazard rate (HR) 1.12 (95% CI: 1.06, 1.18) *P *< 0.001]. This finding was maintained when restricting the analysis to the younger cohort but not the older cohort, with no statistically significant difference in the hazard rate for discontinuation between TNFi monotherapy and TNFi MTX combination (Fig. 2).

When examining TNFi discontinuation by cause, patients in the ≥75 cohort receiving TNFi monotherapy were 34% less likely to discontinue TNFi due to inefficacy compared with patients receiving TNFi MTX combination [HR 0.66 (0.43–0.99) *P *= 0.04]. This finding was not seen in the younger cohort. Patients <75 years old receiving TNFi monotherapy were 6% more likely to discontinue TNFi due to inefficacy compared with patients receiving TNFi MTX, although this was not statistically significant. When examining TNFi discontinuation due to adverse events, patients in both age groups were more likely to discontinue therapy when prescribed TNFi monotherapy compared with TNFi MTX combination [≥75 HR 1.41 (1.02–1.96) *P *= 0.04] and [<75 HR 1.21 (1.11–1.32) *P *< 0.001] (Table 2 and Fig. 2).

There were no meaningful differences in point estimates from complete case analysis and those obtained using the imputed data (Supplementary Table S2, available at *Rheumatology* online). All results remained significant in the multivariable analyses. The PS model also had minimal influence on the point estimates, but the confidence included 1, indicating there may not sufficient evidence to conclude the observed difference is reliable in the over-75s (Supplementary Table S3, available at *Rheumatology* online).

Analyses investigating other TNFi/csDMARD combinations identified a greater risk of discontinuing TNF blockade in the <75 cohort if co-prescribed leflunomide compared with MTX [all cause: adjHR 1.22 (1·08–1.38) *P *= 0.001, and adverse event: adjHR 1.36 (1.13–1.63) *P *= 0.001]. Patients in this younger cohort were also less likely to discontinue anti-TNF if co-prescribed two csDMARDs compared with MTX alone [all cause: adjHR 0.86 (0.79–0.94) *P *<0.001, and adverse event: adjHR 0.85 (0.74–0.98) *P* = 0.02] (Supplementary Table S5, available at *Rheumatology* online).

## Discussion

To our knowledge, this is the first study to investigate drug survival rates with TNFi monotherapy compared with TNFi/csDMARD combination therapy in older adults. In this large observational cohort of 15 000 patients, TNFi monotherapy is associated with an increase in treatment failure. However, in older adults (≥75 years) the disadvantage of TNFi monotherapy on drug survival is no longer seen. This is explained by fewer discontinuations due to inefficacy, but a greater risk of discontinuations due to adverse events. This could be interpreted as evidence that monotherapy is more acceptable in the elderly. An alternative narrative would be that we are observing a phenomenon of ‘competing risks’, an elderly patient may suffer an adverse event leading to termination of therapy, which removes the patient from the ‘risk pool’ prior to the outcome of interest, in this case, loss of drug efficacy.

We also demonstrated significant differences between csDMARD combination strategies. The use of two csDMARDs with TNF blockade is associated with improved drug survival in the younger cohort. However, the cohort was overwhelmingly made up of patients receiving MTX and/or sulfasalazine. Leflunomide was less frequently used, but its presence either alone or in combination had a negative association with TNF inhibitor drug survival, irrespective of age groups.

There are several possible explanations for our findings. Crucially, the adverse event signal seen with TNFi monotherapy compared with TNFi MTX combination therapy may be driven by channelling bias. Channelling is a form of selection bias seen in observational studies, where drugs with similar therapeutic indications are prescribed to groups of patients with prognostic differences [33]. It is plausible that patients with a greater risk of adverse events are more likely to be prescribed TNFi monotherapy which is presumed to have a better safety profile than combination therapy. To address for channelling bias in this cohort, a PS model was created. The technique allows the comparison of non-randomised treatment strategies, adjusting for known covariates that may predict treatment decisions. Despite this, unmeasured confounding likely remains.

In the ≥75-year-old cohort, the lower incidence of failure due to inefficacy with TNFi monotherapy is interesting and potentially of clinical relevance. This may reflect our *a priori* hypothesis that there is a reduction in immunogenicity in this age group, as the aging immune system becomes less effective at mounting antibody responses, as phenomenon known as immunosenescence [34]. Immunogenicity is a recognised mechanism underlying therapeutic failure with TNFi agents over time. Anti-drug antibodies are produced by the immune system in response to proteinaceous drugs, particularly monoclonal antibodies [35,36]. Concomitant use of MTX reduces the clearance of TNFi by lowering the incidence of anti-drug antibodies, resulting in a higher systemic exposure and improved drug survival. In the older cohort, a reduction in immunogenicity may improve TNFi drug survival and preclude the need for concomitant MTX. In support of this immunosenescence hypothesis, the reduced risk of TNFi discontinuation due to inefficacy in patients receiving monotherapy was no longer apparent in the exploratory analyses using a younger age cut-off of 65 and 70.

It is important to note that in our multivariate adjusted analyses, the imputed model demonstrated a statistically significant difference between the TNFi monotherapy and TNFi MTX combination, suggesting that the observed difference is not solely attributable to the measured confounders. However, in the imputed model with PS adjustment, the estimate was non-significant for the over 75s, though the difference in the point estimate between the two models was negligible. A plausible explanation for this is that there is confounding by indication. It is important to acknowledge that our adjustment model includes age and we may be including a path variable if our immunosenescence hypothesis is correct. It remains clear that age or some mechanism related to age is likely to be important in explaining the difference in effect of TNFi monotherapy *vs* combination therapy.

The effect size (adjusted hazard ratio of 0.63) suggests patients ≥75 receiving TNFi monotherapy are nearly 40% less likely to discontinue TNFi due to inefficacy compared with patients receiving TNFi MTX combination. In part, this may be explained by the competing risk phenomenon; some patients who were destined to fail due to inefficacy experience an adverse event before meeting the inefficacy end point, thereby selecting themselves out of the ‘at risk of inefficacy’ cohort. Older patients are more likely to stop TNFi therapy than younger patients, and adverse events is the highest contributing reason for discontinuation. This may explain the slightly paradoxical finding that fewer older people stop due to inefficacy on monotherapy. The finding of higher discontinuation rates in the elderly is not surprising. Age is a consistent predictor for many outcomes that may lead to discontinuation, such as infection or cancer and direct drug toxicity.

Our results are in keeping with published data from observational studies. The Dutch and Swiss registries reported comparable drug survival and reasons for discontinuations between the young and the elderly [14, 16], while the Italian registry demonstrated greater discontinuation in the elderly, with more frequent adverse events [17]. Zhang *et al.* demonstrated that concomitant MTX improves persistence to biologic therapy in patients over 65 years, although analyses included patients <65 years old with certain disabilities, and no information was provided regarding reasons for discontinuation [37]. In contrast to earlier analyses using BSRBR data, we did not demonstrate inferiority of the sulfasalazine/TNFi combination [9]. We did however confirm the association with leflunomide and lower TNFi treatment survival, which has also been demonstrated in the German registry, although this did not reach statistical significance [38].

This study has several strengths. The large sample size, limited missing data and accurate coding of treatment discontinuation has facilitated an in-depth and robust analysis. The BSRBR-RA includes data on elderly patients who are frequently excluded from clinical trials and provides real world data improving generalisability to clinical practice.

Despite the large overall sample size, the size of the ≥75-year cohort was relatively small, particularly in the ‘inefficacy’ model that limits statistical power. The decision to stop anti-TNF therapy and the reason for discontinuation was provided by the supervising rheumatologist, and we are unable to externally verify the accuracy of data provided. This may account for the number of ‘other’ or ‘missing’ entrees, possibly introducing a degree of misclassification bias. All our analyses were based on csDMARD regimen at study entry. Patients may modify their csDMARD regimen after the introduction of TNFi. During the 5-year observation period, 18% of the cohort changed from their initial therapy choice of TNFi monotherapy, TNFi MTX combination or TNFi-other csDMARD combination. Six percent of the cohort switched between TNFi monotherapy and TNFi MTX combination. The proportion of ‘switchers’ was similar between the two age cohorts. We did not consider patients who switched between initial therapy choice in our analyses and this may have influenced TNFi survival. We chose to exclude previous csDMARD exposure from our multivariate model despite recognising this as an important confounder. This is because prior csDMARD therapy associates with our predictor variable (i.e. being on TNFi monotherapy is more likely to be associated with multiple failed csDMARDs). Lastly, in this analysis we tested multiple hypotheses, which potentially increases the chances of a false-positive association, and as such our results should be interpreted with caution. Replicating these analyses in other registries’ data and corroborating our results would prove invaluable.

In conclusion, these data provide evidence to support TNFi monotherapy strategies in the over-75s in the wider context of a desire to reduce polypharmacy burden, the findings in this study should help alleviate physician concerns about drug immunogenicity in older patients.

## Supplementary Material

kez671_Supplementary_Data
