# Corrigendum to: Is background methotrexate advantageous in extending TNF inhibitor drug survival in elderly patients with rheumatoid arthritis? An analysis of the British Society for Rheumatology Biologics Register

**DOI:** 10.1093/rheumatology/keaa612

**Published:** 2020-10-07

**Authors:** Katie Bechman, Anuoluwapo Oke, Mark Yates, Sam Norton, Elaine Dennison, Andrew P Cope, James B Galloway

**Affiliations:** 1 Centre for Rheumatic Diseases, Kings College London, London, UK; 2 MRC Lifecourse Epidemiology Unit, University of Southampton, Southampton, UK; 3 Psychology Department, Institute of Psychiatry, Kings College London, London, UK

Rheumatology 2020;59:2563–2571

doi:10.1093/rheumatology/kez671

In the above article, image C and D in Figure 2 was originally a duplicate of image A and B. This has been corrected online and in print. The author apologises for the error.

