# Supplementary material for: Is background methotrexate advantageous in extending TNF inhibitor drug survival in elderly patients with rheumatoid arthritis? An analysis of the British Society for Rheumatology Biologics Register
Source: Rheumatology (Oxford). 2020 Jan 30;59(9):2563–71. doi: 10.1093/rheumatology/kez671 (PMC7449803; doi:10.1093/rheumatology/kez671)
Supplement: kez671_Supplementary_Data [file kez671_supplementary_data.docx]

**SUPPLEMENTARY MATERIAL**

**Supplementary Table S1 Incidence rates and Cox proportional hazard estimates (95% CI) for anti-TNF therapy discontinuation by different age cut-off**

| Number of subjects | <65yrs (n=11,850) | ≥65yrs (n=3,850) |
| --- | --- | --- |
| TNF failure – all cause | | |
| No. of patients  Incidence rate per 100 patient years (95% CI)  HZ (95% CI) (ref MTX): Unadjusted; Monotherapy  Adjusted; Monotherapy | 6098  17.6 (17.1, 18.0)  1.03 (0.97-1.10)  1.00 (0.93-1.08) | 2108  21.3 (20.4, 22.2)  1.32 (1.19-1.47) *  1.28 (1.13-1.44) * |
| TNF failure – inefficacy | | |
| No. of patients  Incidence rate per 100 patient years (95% CI)  HZ (95% CI) (ref MTX): Unadjusted; Monotherapy  Adjusted; Monotherapy | 2581  7.4 (7.1, 7.7)  1.00 (0.91-1.11)  0.95 (0.84-1.07) | 761  7.7 (7.2, 8.2)  1.16 (0.97-1.38)  1.09 (0.89-1.34) |
| TNF failure – adverse events | | |
| No. of patients  Incidence rate per 100 patient years (95% CI)  HZ (95% CI) (ref MTX): Unadjusted; Monotherapy  Adjusted; Monotherapy | 2295  6.6 (6.3, 6.9)  1.10 (0.99-1.22)  1.08 (0.95-1.22) | 958  9.7 (9.1, 10.3)  1.49 (1.28-1.73) *  1.45 (1.22-1.73) * |
| Number of subjects | <70yrs (n=13,777) | ≥70yrs (n=1,923) |
| TNF failure – all cause | | |
| No. of patients  Incidence rate per 100 patient years (95% CI)  HZ (95% CI) (ref MTX): Unadjusted; Monotherapy  Adjusted; Monotherapy | 7111  17.8 (17.4, 18.2)  1.08 (1.02-1.15) *  1.05 (0.98-1.12) | 1095  23.2 (21.9, 24.6)  1.28 (1.11-1.48) *  1.27 (1.07-1.50) * |
| TNF failure – inefficacy | | |
| No. of patients  Incidence rate per 100 patient years (95% CI)  HZ (95% CI) (ref MTX): Unadjusted; Monotherapy  Adjusted; Monotherapy | 2968  7.4 (7.2, 7.7)  1.05 (0.96-1.15)  1.00 (0.90-1.12) | 374  7.9 (7.2, 8.8)  0.96 (0.74-1.22)  0.90 (0.67-1.22) |
| TNF failure – adverse events | | |
| No. of patients  Incidence rate per 100 patient years (95% CI)  HZ (95% CI) (ref MTX): Unadjusted; Monotherapy  Adjusted; Monotherapy | 2739  6.9 (6.6, 7.1)  1.17 (1.07-1.29) *  1.14 (1.02-1.27) † | 514  10.9 (10.0, 11.9)  1.45 (1.18-1.79) *  1.48 (1.17-1.88) * |

Adjusted for age, gender, disease duration, Rheumatic Disease Comorbidity Index, smoking, DAS28, HAQ-DI and steroid use. Reference group: TNFi-Methotrexate combination. Supplementary Table S5 reports on the hazard estimates for TNFi discontinuation by choice of combination therapy including TNFi-sulfasalazine, TNFi-leflunomide or TNF-multiple csDMARDs.

TNFi: TNF inhibitor; *: p-value <0.01; †: p-value <0.05

**Imputation methodology**

The predictor and outcome data were near complete; only 1 patient did not have a recorded age. There were missing data for several baseline variables used in the multivariate analysis. Data on gender, comorbidity and steroid use were complete. Missing data is presented below. The below variables with incomplete data were imputed. All missing data were imputed regardless of the reason or reasons it was missing. The following variables with complete data were utilised for the imputation: age; gender; comorbidity; steroid use; previous DMARDs exposure, current DMARDs therapy, choice of TNF therapy, time to TNF therapy discontinuation and reason for discontinuation. Linear and logistic regression were performed to impute the normally distributed and dichotomous variables respectively. The data were imputed using multivariate sequential imputation using chained equations. Firstly, all missing values were filled in by simple random sampling with replacement from the observed values. The first variable with missing values was regressed on all other variables. The imputation was 20 cycles, where at the end of the cycle one imputed dataset was created and the process was repeated to create 20 imputed datasets. The 20 datasets were combined using Rubin’s rules, therefore the estimates and standard errors presented here are the combined ones.

**Supplementary Table S2a Missing data**

| Variable | Observations | Missing Variables |
| --- | --- | --- |
| Age | 15699 | 1 |
| Disease duration | 15,537 | 163 |
| DAS28-ESR | 14,997 | 703 |
| HAQ-DI | 12,659 | 3041 |
| Smoking status | 12,986 | 2741 |
| Seropositive | 15,140 | 560 |

**Supplementary Table S2b Differences between the results obtained from complete case analysis and those obtained using multiply imputed data.**

| Number of subjects | <75yrs | ≥75yrs | Total |
| --- | --- | --- | --- |
| TNF failure – all cause (reference methotrexate) | | | |
| Complete case analysis (unimputed dataset)   - Unadjusted Monotherapy - Adjusted; Monotherapy | - 1. (1.05-1.17) *   1.08 (1.01-1.15) † | 1.13 (0.90-1.41)  1.16 (0.89-1.52) | 1.12 (1.06-1.18) *  1.08 (1.01-1.15) * |
| Multiply imputed data   - Adjusted; Monotherapy | 1.07 (1.01-1.13) * | 1.15 (0.91-1.45) | 1.08 (1.02-1.14) * |
| TNF failure – inefficacy (reference methotrexate) | | | |
| Complete case analysis (unimputed dataset)   - Unadjusted Monotherapy - Adjusted; Monotherapy | 1.06 (0.97-1.16)  1.01 (0.91-1.12) | 0.66 (0.43-0.99) †  0.59 (0.35-0.97) † | 1.03 (0.95-1.13)  0.99 (0.89-1.09) |
| Multiply imputed data   - Adjusted; Monotherapy | 1.06 (0.97-1.16) | 0.63 (0.41-0.97) † | 1.03 (0.94-1.13) |
| TNF failure – adverse events (reference methotrexate) | | | |
| Complete case analysis (unimputed dataset)   - Unadjusted Monotherapy - Adjusted; Monotherapy | 1.21 (1.11-1.32) *  1.17 (1.05-1.30) * | 1.41 (1.02-1.96) †  1.63 (1.10-2.40) † | 1.23 (1.13-1.34) *  1.19 (1.08-1.31) * |
| Multiply imputed data   - Adjusted; Monotherapy | 1.13 (1.03-1.23) * | 1.46 (1.05-2.03) † | 1.14 (1.05-1.25) * |

Adjusted for age, gender, disease duration, Rheumatic Disease Comorbidity Index, smoking, DAS28, HAQ-DI and steroid use. Reference group: TNFi-Methotrexate combination. Supplementary Table S5 reports on the hazard estimates for TNFi discontinuation by choice of combination therapy including TNFi-sulfasalazine, TNFi-leflunomide or TNF-multiple csDMARDs. TNFi: TNF inhibitor; *: p-value <0.01; †: p-value <0.05.

**Propensity Model methodology**

A single-variable logistic regression model was used to identify baseline covariates that predicted treatment choice (monotherapy versus methotrexate combination therapy). A multivariable logistic regression model using significant predictors was used to create a single propensity score for each individual. A Hosmer-Lemeshow test was used to assess the regression equation. A propensity score model was created including the following covariates: age, gender, disease duration, RDCI, DAS28, HAQ, smoking status and steroid exposure.

The inverse of the probability (or the inverse of 1 minus the probability in the monotherapy cohort) was then used as the treatment weight in the analysis. Truncation of weights was used to prevent a small number of larger weights de-stabilising the model. The balancing of the cohorts using the weighted model was tested by comparing standardised differences between cohorts. The weighted means and standard differences are shown below.

**Supplementary Table S3a: Comparison of Baseline Covariates in Weighted Cohorts**

|  | Mean in TNF Monotherapy | Mean in TNF-MTX combination | Standardised difference |
| --- | --- | --- | --- |
| Before weighting | | | |
| Baseline Age (years)  Female  RDCI Score  Disease Duration in years  Baseline DAS28 Score  Baseline HAQ Score  Steroid users  Smoker | 57.82  0.76  1.15  14.93  6.61  2.111  0.51  0.63 | 54.88  0.75  0.98  13.40  6.49  1.99  0.40  0.61 | 0.236  0.025  0.149  0.156  0.118  0.209  0.231  0.044 |
| After weighting – unimputed baseline data | | | |
| Baseline Age (years)  Female  RDCI Score  Disease Duration in years  Baseline DAS28 Score  Baseline HAQ Score  Steroid users  Smoker | 55.82  0.75  1.04  13.99  6.52  2.03  0.44  0.62 | 55.95  0.76  1.04  13.99  6.53  2.04  0.44  0.62 | -0.010  -0.008  -0.002  0.000  -0.011  -0.008  -0.005  -0.003 |
| After weighting - imputed baseline data | | | |
| Baseline Age (years)  Female  RDCI Score  Disease Duration in years  Baseline DAS28 Score  Baseline HAQ Score  Steroid users  Smoker | 56.41  0.77  1.05  14.16  6.55  2.06  0.46  0.62 | 55.75  0.75  1.05  13.93  6.52  2.03  0.43  0.62 | 0.048  0.060  0.002  0.023  0.034  0.020  0.003  -0.052 |

TNFi: TNF inhibitor; RDCI: Rheumatic Disease Comorbidity Index; DAS28: Disease Activity Score 28 Joints; *: p-value <0.01. †: p-value <0.05.

**Supplementary table S3b Propensity score model: Cox proportional hazard estimates (95% CI) for anti-TNF therapy discontinuation**

| Number of subjects | Age | | |
| --- | --- | --- | --- |
|  | <75yrs | ≥75yrs | Total |
| TNF failure – all cause (reference methotrexate) | | | |
| Primary analysis   - Unadjusted Monotherapy - Adjusted; Monotherapy | - 1. (1.05-1.17) *   1.08 (1.01-1.15) † | 1.13 (0.90-1.41)  1.16 (0.89-1.52) | 1.12 (1.06-1.18) *  1.08 (1.01-1.15) * |
| Propensity score model   - Complete case analysis (unimputed dataset) - Multiply imputed data | 1.05 (0.98-1.13)  1.06 (1.00-1.12) † | 1.15 (0.89-1.49)  1.12 (0.90-1.40) | 1.06 (0.99-1.13)  1.06 (1.01-1.13) † |
| TNF failure – inefficacy (reference methotrexate) | | | |
| Primary analysis   - Unadjusted Monotherapy - Adjusted; Monotherapy | 1.06 (0.97-1.16)  1.01 (0.91-1.12) | 0.66 (0.43-0.99) †  0.59 (0.35-0.97) † | 1.03 (0.95-1.13)  0.99 (0.89-1.09) |
| Propensity score model   - Complete case analysis (unimputed dataset) - Multiply imputed data | 1.01 (0.91-1.12)  1.06 (0.97-1.16) | 0.65 (0.41-1.05)  0.69 (0.45-1.04) | 0.99 (0.89-1.10)  1.04 (0.95-1.13) |
| TNF failure – adverse events (reference methotrexate) | | | |
| Primary analysis   - Unadjusted Monotherapy - Adjusted; Monotherapy | 1.21 (1.11-1.32) *  1.17 (1.05-1.30) * | 1.41 (1.02-1.96) †  1.69 (1.10-2.40) † | 1.23 (1.13-1.34) *  1.19 (1.08-1.31) * |
| Propensity score model   - Complete case analysis (unimputed dataset) - Multiply imputed data | 1.14 (1.03-1.27) †  1.11 (1.02-1.22) * | 1.50 (1.03-2.20) †  1.35 (0.97-1.88) | 1.16 (1.04-1.28) *  1.13 (1.04-1.23) * |

Adjusted for age, gender, disease duration, Rheumatic Disease Comorbidity Index, smoking, DAS28, HAQ-DI and steroid use. Reference group: TNFi-Methotrexate combination. TNFi: TNF inhibitor; *: p-value <0.01; †: p-value <0.05

**Supplementary Table S4. Baseline table comparing patients on combination csDMARD and TNFi versus patients prescribed TNFi monotherapy**

|  | Combination therapy | Monotherapy | Stat. imbalance |
| --- | --- | --- | --- |
| Total cohort, n (%) | 11, 790 (75.1) | 3910 (24.9) |  |
| Age, yrs., mean (SD) | 55 (46-64) | 58 (48-66) | <0.0001* |
| Female sex, n (%) | 8605 (73.0) | 2810 (71.9) | 0.17 |
| Smoking status, n (%)   - Current - Ever | 1997 (21.5)  5917 (61.0) | 694 (21.8)  2073 (63.0) | 0.65^†^  0.05^†^ |
| Comorbidity (RDCI score ≥1)   - Cardiac (MI, stroke, angina) - Respiratory (asthma, COPD) | 6467 (54.9)  764 (6.5)  1615 (13.7) | 2387 (61.1)  337 (8.6)  594 (15.2) | <0.001^†^  <0.001^†^  <0.02^†^ |
| Seropositive (RF), n (%) | 6794 (59.2) | 2128 (58.2) | <0.29^†^ |
| Disease duration, yrs. | 10 (5-17) | 12 (6-21) | <0.0001* |
| Number of previous DMARDs | 3 (2-4) | 4 (3-5) | <0.0001* |
| TNF, n (%)   - Infliximab - Etanercept - Adalimumab - Certolizumab | 3882 (32.9)  3349 (28.4)  3791 (32.2)  768 (6.5) | 282 (7.2)  2290 (58.6)  1199 (30.7)  139 (3.6) | <0.001^†^ |
| Prednisolone, n (%) | 4449 (37.7) | 1819 (46.5) | <0.001^†^ |
| DAS28-ESR, mean (SD) | 6.39 (1.0) | 6.54 (1.1) | <0.001* |
| SJC28, mean (SD) | 10.7 (6.1) | 10.8 (6.3) | 0.36* |
| TJC28, mean (SD) | 15.1 (7.4) | 15.5 (7.8) | 0.03* |
| Global VAS | 75 (60-85) | 78 (64-90) | <0.0001* |
| ESR | 36 (21-60) | 42 (25-67) | <0.0001* |
| CRP mg/l | 25 (11-53) | 29 (12-65) | <0.0001* |
| HAQ-DI, median (IQR) | 2 (1.625, 2.375) | 2.25 (1.75, 2.5) | <0.0001* |

All values are gives as median (IQR), unless otherwise specified by n(%) or mean (SD). Statistical imbalance tested or χ2 † or kwallis*. TNFi: TNF inhibitor; RDCI: Rheumatic Disease Comorbidity Index; DAS28: Disease Activity Score 28 Joints; SJC28: 28 swollen joint count; TJC28: 28 tender joint count; Global VAS: visual analogue scale for patient’s global assessment.

**Supplementary Table S5 Incidence rates and Cox proportional hazard estimates (95% CI) for anti-TNF therapy discontinuation by choice of combination therapy**

| TNF failure – all cause | <75yrs | ≥75yrs | Total |
| --- | --- | --- | --- |
| Follow up (Person-years)/100 | 42876 | 1766 | 44642 |
| No. of TNF patients with TNF failures | 7756 | 450 | 8206 |
| Incidence rate per 100 patient years (95% CI) | 18.1 (17.7-18.5) | 25.5 (23.2-27.9) | 18.4 (18.0-18.8) |
| Unadjusted HR (95% CI) - MTX (reference)   - Monotherapy - Sulfasalazine - Leflunomide - Two csDMARDs - Three csDMARDs | 1.11 (1.05-1.17) *  0.95 (0.82-1.09)  1.23 (1.11-1.37) *  0.82 (0.76-0.87) *  0.90 (0.80-1.01) | 1.13 (0.90-1.41)  1.48 (0.87-2.53)  1.32 (0.88-1.98)  0.91 (0.66-1.24)  1.04 (0.62-1.74) | 1.12 (1.06-1.18) *  0.97 (0.85-1.11)  1.24 (1.12-1.38) *  0.82 (0.77-0.88) *  0.90 (0.81-1.01) |
| Adjusted HR (95% CI) - MTX (reference)   - Monotherapy - Sulfasalazine - Leflunomide - Two csDMARDs - Three csDMARDs | 1.08 (1.01-1.15) †  1.01 (0.85-1.20)  1.22 (1.08-1.38) *  0.86 (0.79-0.94) *  0.96 (0.84-1.11) | 1.17 (0.90-1.53)  1.85 (0.99-3.48)  1.40 (0.89-2.18)  1.08 (0.74-1.57)  0.93 (0.47-1.85) | 1.08 (1.01-1.15) *  1.04 (0.88-1.23)  1.23 (1.10-1.39) *  0.88 (0.81-0.95) *  0.97 (0.85-1.11) |
| TNF failure – inefficacy | | | |
| Follow up (Person-years) | 42876 | 1766 | 44642 |
| No. of TNF patients with TNF inefficacy | 3193 | 149 | 3342 |
| Incidence rate per 100 patient years (95% CI) | 7.45 (7.19-7.71) | 8.44 (7.18-9.91) | 7.49 (7.24-7.74) |
| Unadjusted HR (95% CI) - MTX (reference)   - Monotherapy - Sulfasalazine - Leflunomide - Two csDMARDs - Three csDMARDs | 1.06 (0.97-1.16)  0.98 (0.79-1.22)  1.18 (1.00-1.39)  0.94 (0.84-1.04)  0.95 (0.79-1.13) | 0.66 (0.43-0.99) †  1.10 (0.45-2.68)  0.88 (0.42-1.86)  1.08 (0.67-1.74)  1.23 (0.55-2.76) | 1.03 (0.95-1.13)  0.99 (0.80-1.22)  1.16 (0.99-1.36)  0.94 (0.85-1.04)  0.96 (0.80-1.14) |
| Adjusted HR (95% CI) - MTX (reference)   - Monotherapy - Sulfasalazine - Leflunomide - Two csDMARDs - Three csDMARDs | 1.01 (0.91-1.12)  1.01 (0.77-1.32)  1.16 (0.96-1.40)  0.93 (0.82-1.05)  0.97 (0.78-1.20) | 0.59 (0.35-0.97) †  1.77 (0.72-4.37)  0.85 (0.39-1.86)  1.15 (0.64-2.07)  1.15 (0.38-3.41) | 0.99 (0.89-1.09)  1.04 (0.80-1.35)  1.14 (0.95-1.38)  0.94 (0.83-1.07)  0.98 (0.80-1.20) |
| TNF failure – adverse event | | | |
| Follow up (Person-years) | 42876 | 1766 | 44642 |
| No. of TNF patients with TNF inefficacy | 3044 | 209 | 3253 |
| Incidence rate per 100 patient years (95% CI) | 7.10 (6.85-7.36) | 11.83 (10.33-13.55) | 7.29 (7.04-7.54) |
| Unadjusted HR (95% CI) - MTX (reference)   - Monotherapy - Sulfasalazine - Leflunomide - Two csDMARDs - Three csDMARDs | 1.21 (1.11-1.32) *  0.99 (0.79-1.23)  1.35 (1.15-1.59) *  0.74 (0.66-0.83) *  0.82 (0.68-1.00) | 1.41 (1.02-1.96) †  1.53 (0.68-3.44)  1.38 (0.75-2.55)  0.82 (0.50-1.33)  0.85 (0.37-1.93) | 1.23 (1.13-1.34) *  1.02 (0.82-1.26)  1.36 (1.16-1.59) *  0.74 (0.66-0.83) *  0.82 (0.68-1.00) † |
| Adjusted HR (95% CI) - MTX (reference)   - Monotherapy - Sulfasalazine - Leflunomide - Two csDMARDs - Three csDMARDs | 1.17 (1.06-1.30) *  1.11 (0.84-1.44)  1.36 (1.13-1.63) *  0.85 (0.74-0.98) †  1.02 (0.81-1.28) | 1.64 (1.11-2.42) †  1.42 (0.53-3.78)  1.66 (0.85-3.24)  1.12 (0.65-1.93)  0.67 (0.22-2.03) | 1.19 (1.08-1.32) *  1.11 (0.86-1.43)  1.37 (1.15-1.64) *  0.86 (0.75-0.98) †  1.00 (0.80-1.25) |

Adjusted for age, gender, disease duration, Rheumatic Disease Comorbidity Index, smoking, DAS28, HAQ-DI and steroid use. Reference group: TNFi-Methotrexate combination. Supplementary Table S5 reports on the hazard estimates for TNFi discontinuation by choice of combination therapy including TNFi-sulfasalazine, TNFi-leflunomide or TNF-multiple csDMARDs. TNFi: TNF inhibitor; *: p-value <0.01; †: p-value <0.05
